# Supplementary material for: The CellPhe toolkit for cell phenotyping using time-lapse imaging and pattern recognition
Source: Nat Commun. 2023 Apr 3;14:1854. doi: 10.1038/s41467-023-37447-3 (PMC10070448; doi:10.1038/s41467-023-37447-3)
Supplement: Supplementary file 1 — Supplementary Information [file 41467_2023_37447_MOESM1_ESM.pdf]

Supplementary Information

1 Supplementary notes

1.1 Feature extraction

Supplementary Table 1 shows the features extracted for each cell on each frame. As the 14 Haralick features are calculated from each of 3 co-occurrence matrices to give 42 texture features, a total of 74 features are calculated for each cell on each frame. The time series for each of these features is summarised by three statistical variables as well as three variables calculated from each of the original time series and three levels of the wavelet-transformed time series, as shown in Supplementary Table 2, giving 15 variables for each extracted feature. One final variable, the area of the rectangle enclosing the cell's trajectory, is calculated to give a total of 1111 variables. Note that, five of these variables cannot be discriminatory and are not included in any analysis or classification: the total descent for the feature *tracklength* will always be zero (4 variables) and, as the tracklength and displacement are the same for the first frame and the displacement can never be greater than the tracklength, the maximum value for their quotient will always be 1. These variables are not removed as they will never be chosen by the feature selection algorithm but users wanting to perform their own analysis on the full set of variables would need to remove them before, for example, scaling the data.

We concatenate feature and variable names throughout for brevity. Variables extracted from raw time series are denoted in the form "feature_variable", for example "Volume_mean" denotes a cell's mean volume throughout its track. Variables extracted from wavelet approximated time series are denoted in the form "feature_level_variable", for example "Volume_l1_mean", "Volume_l2_mean", "Volume_l3_mean" denote a cell's mean volume at the first, second and third wavelet approximation respectively. The Haralick features are calculated from three co-occurrence matrices: the original image vs. level 1 wavelet approximation, the original image vs. level 2 wavelet approximation, and level 1 wavelet approximation vs. level 2 wavelet approximation, denoted 01, 02 and 12 respectively. Hence Haralick features are concatenated with extracted variables in the form "feature_cooccurrence_variable", for example "Hf1_01_mean" denotes a cell's mean energy extracted from the original image vs. level 1 wavelet approximation co-occurrence matrix.

1.2 Cluster analysis

Our cell type classification results demonstrated a class imbalance in accuracy scores, with a greater number of untreated MCF-7 classified as treated in comparison to treatment classification results involving MDA-MB-231 cells. Further analysis of the untreated MCF-7 cells identified a subpopulation of instances of cell debris that were misclassified as treated cells, adding to the proportion of untreated cell misclassifications. Cluster analysis of the untreated MCF-7 set is provided in Supplementary Figure 1.

1.3 Dose response experiments

To confirm the ability of our classification models to identify a population of cells that do not respond to drug treatment, we classified cells that received varying concentrations of drug. As expected, a greater number of treated cells were classified as untreated at lower drug concentrations

in comparison to higher concentrations where a greater number of cells responded to drug. Representative time-lapse images of cells from a single experiment in which individual wells were treated with varying concentrations of drug are provided in Supplementary Figures 2-4.

2 Supplementary tables

**Feature**	**Short name**	**Type**	**Description**
Velocity	vel	Movement1	Current relative speed
Displacement	dis	Movement2	Euclidean distance from initial position
Tracklength	trac	Movement3	Path length travelled up to current frame
Displacement fraction of tracklength	D2T	Movement4	Current displacement divided by current tracklength
†Volume	vol	Size1	Cell volume, calculated using phase information
Radius	rad	Size2	Average distance of boundary pixels from centroid
Length	len	Size3	Maximum vertical or horizontal distance across the cell
Width	wid	Size4	Maximum distance across the cell perpendicular to Length.
Area	area	Size5	The number of pixels within the cell
†Sphericity	sph	Shape1	Sphericity calculated using phase information
Minimal box	box	Shape2	Fraction of minimal bounding box containg cell pixels
Rectangularity	rect	Shape3	Measure of cell elongation
Variance from centroid	VfC	Shape4	Variance in distance to boundary pixels from cell centroid.
Curvature	cur	Shape5	Measure of boundary curvature based on the triangle inequality.
Area to boundary ratio	A2B	Shape6	The number of cell pixels divided by the number of boundary pixels squared
Shape descriptors from polygonal approximation to cell (4)	poly1	Shape7	Mean edge length
	poly2	Shape8	Variance in edge length
	poly3	Shape9	Mean interior angle
	poly4	Shape10	Variance in interior angle
First order features from cell interior pixels (3)	FOf1	Texture1	Mean of pixel intensities
	FOf2	Texture2	Standard deviation of pixel intensities
	FOf3	Texture3	Skewness of pixel intensities

**Feature**	**Short name**	**Type**	**Description**
Spatial distribution descriptors (9)	IQ1 to IQ9	Texture1 to Texture9	Measure of dispersion for pixel intensities in different quantiles
Haralick features from co-occurrence matrix (3×14)	Hf1 to Hf14	Texture10 to Texture51	Angular Second Moment; Contrast; Correlation; Variance; Homogeneity; Sum Average; Sum Variance; Entropy; Sum Entropy; Difference Variance; Difference Entropy; Information Measure of Correlation 2; Cluster Shade; Cluster Prominence
Density	den	Density1	The local density calculated as the sum of inverse distances from the cell centroid to those of neighbouring cells within three times the cells diameter.

Supplementary Table S1: Table of features extracted from each cell on each frame. Where more than one feature of a particular type is produced, the number of features is shown in parentheses.

**Variable**	**short name**	**Description**
Mean<br>Standard deviation<br>Skewness	mean<br>std<br>skew	Summary statistics from cell time series
Total ascent<br>Total descent<br>Maximum altitude gain	asc<br>des<br>max	Total increase over time series<br>Total decrease over time series<br>Maximum increase from start of time series
Total ascent	asc	Sum of negative detail coefficients from 3 levels of the wavelet-transformed time series
Total descent	des	Sum of positive detail coefficients from 3 levels of the wavelet-transformed time series
Maximum detail coefficient	max	maximum change between frames
Trajectory area	trajarea	Rectangular area enclosing cell time series

Supplementary Table S2: Table of variables extracted from each cell's time series.

3 Supplementary figures

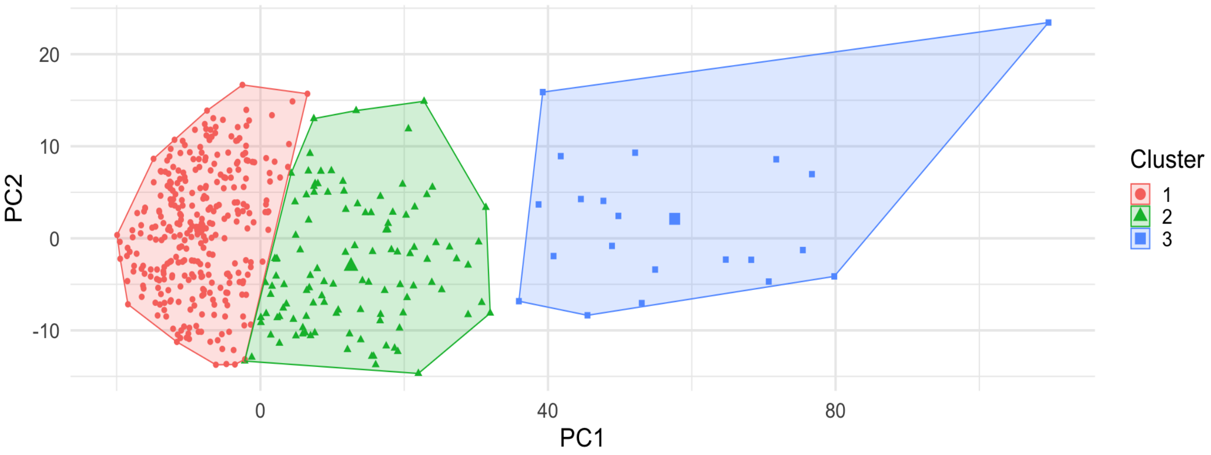

Supplementary Figure 1. Cluster analysis of untreated cells from the MCF7Docetaxel test set. Cluster 3 is formed of cell debris, or cells that share similar texture to cell debris, where 85% of the cells within this cluster were misclassified as treated. Source data are provided in the Source Data file.

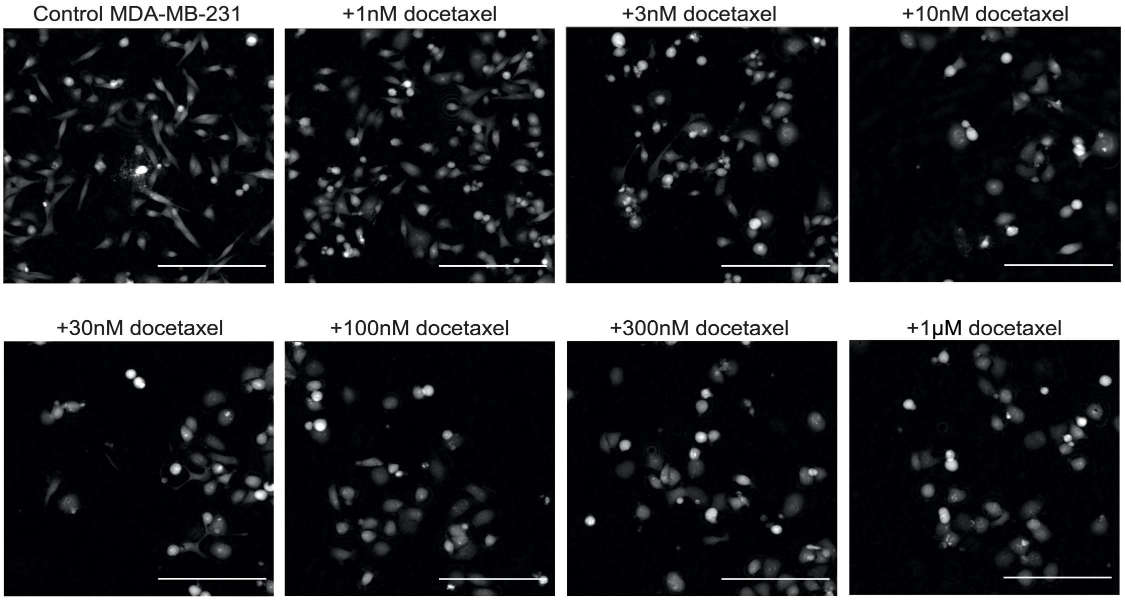

Supplementary Figure 2. Images of MDA-MB-231 cells taken 48 hours after treatment with varying concentrations of docetaxel. Scale bar = 200μm.

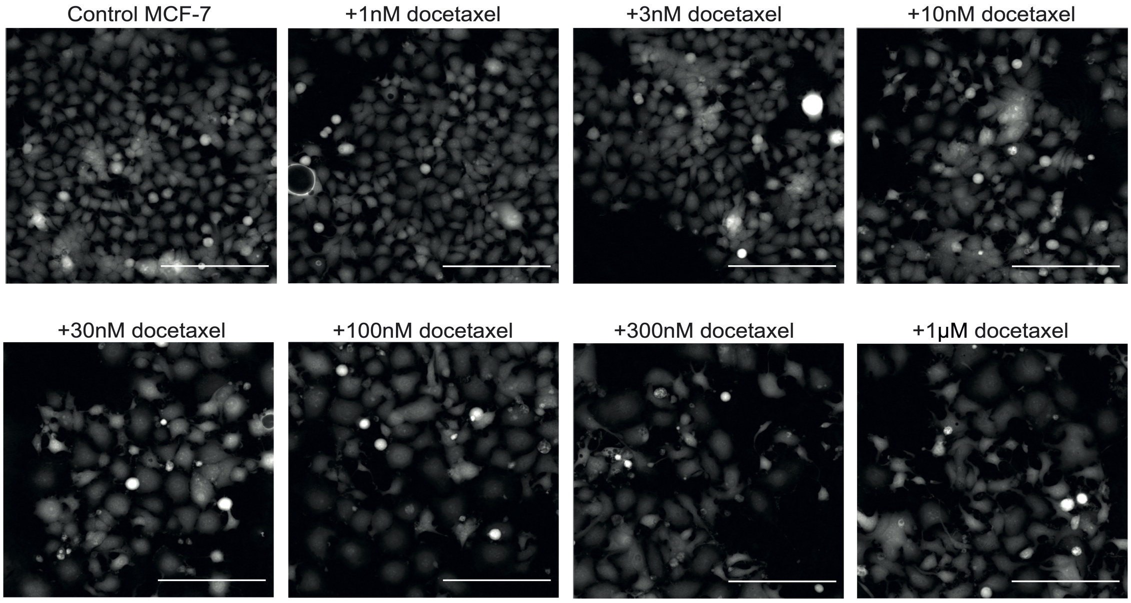

Supplementary Figure 3. Images of MCF-7 cells taken 48 hours after treatment with varying concentrations of docetaxel. Scale bar = 200μm.

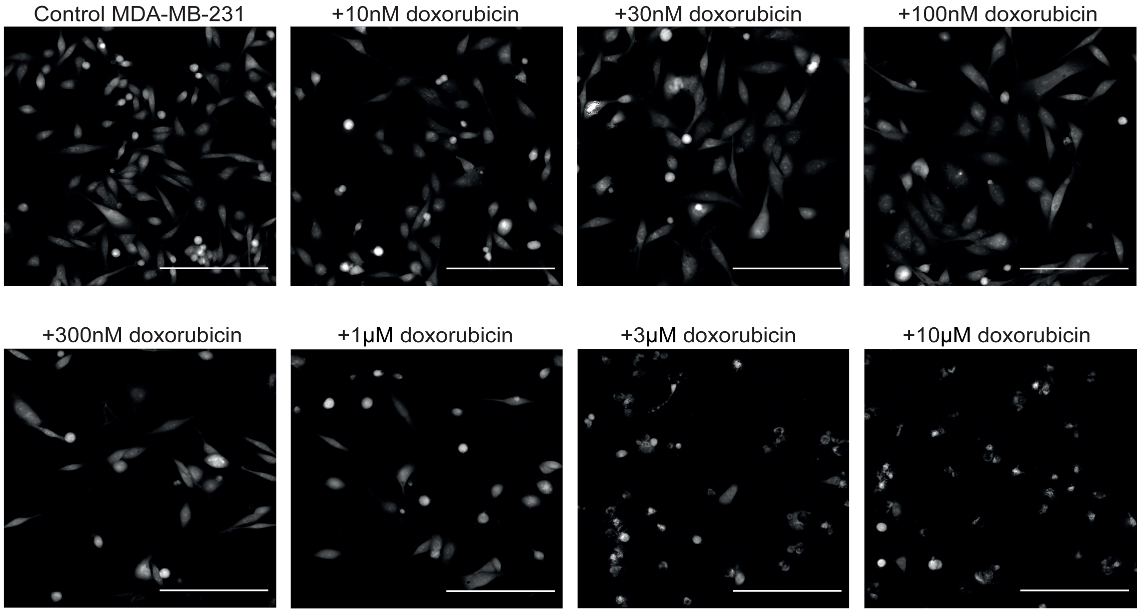

Supplementary Figure 4. Images of MDA-MB-231 cells taken 48 hours after treatment with varying concentrations of doxorubicin. Scale bar = 200μm.

CellPhe user guide

CellPhe is a pattern recognition toolkit for the unbiased characterisation of cellular phenotypes within time-lapse videos. The toolkit is available on GitHub as an R package together with a user-friendly interactive GUI. This manual aims to guide users through the complete CellPhe workflow with a reproducible worked example.

All data used to produce the results in the manuscript, including separate data that will allow the user to follow the worked example in this user guide, are available from Dryad with the digital object identifier (DOI): doi:10.5061/dryad.4xgxd25f0. Here, the file `example_data.zip` contains all the data required to follow the worked example below and the file `CellPhe_GUI_demo_vid.mov` is a video that explains how to use the GUI.

4 CellPhe R package

4.1 Installing CellPhe

After downloading the CellPhe code from https://github.com/uoy-research/CellPhe as a zip file using the green Code tab, the uncompressed file should be renamed as just CellPhe. Install dependencies in R:

```
install.packages(c("tiff", "smotefamily"))
library(tiff)
library(smotefamily)
install.packages(c("randomForest", "e1071", "factoextra", "tree"))
library(randomForest)
library(e1071)
library(factoextra)
library(tree)
```

In a Terminal window, in the directory above the one where the (uncompressed) CellPhe code is, type:

```
$ R CMD build CellPhe
$ R CMD INSTALL CellPhe_0.0.0.9000.tar.gz
```

Alternatively, the R package can be installed directly in R by running the following lines of code:

```
install.packages("devtools")
library(devtools)
install_github("uoy-research/CellPhe")
library(CellPhe)
```

To follow the worked example included in this manual, ensure that the example data sets are within your current R working directory.

The interactive CellPhe GUI can be accessed here: https://cellphegui.shinyapps.io/app_to_host/

4.2 Extraction of time series variables

The function `copyFeatures()` copies the tracking information (frame and cell identifiers) and any chosen features extracted by the tracking software from an input csv file. Currently CellPhe accepts input data from either PhaseFocus or TrackMate software, using `source = "Phase"` or `source = "Trackmate"`. For example, for PhaseFocus generated data, volume and sphericity features are copied as these rely on phase information. Only cells that are tracked for a minimum of `minframes` are included. Output is a dataframe with each row corresponding to a cell tracked on one frame. The first three columns give the `FrameID`, `CellID` and the name of the corresponding ROI file containing the boundary information (as output by either PhaseFocus or TrackMate) which are followed by a column for each copied feature. Note that ROIs must contain full boundary coordinates. Files output by TrackMate only give the coordinates of the vertices of a polygon, but interpolation can be performed to extract full coordinates using ImageJ. An ImageJ macro, with instructions for use, is provided within the CellPhe GitHub repository.

The output from `copyFeatures()` is then read into the function `extractFeatures()` which calculates a further 72 features related to size, shape, texture and movement for each cell on every non-missing frame, as well as the cell density around each cell on each frame. If the frame rate is input by the user then velocity will be calculated using this information (`framerate`), otherwise a default value of 1 will be used, which does not affect the discriminatory power of the variable.

Note that, if the ROI folder is compressed, it should be unzipped and the image folder should contain individual tiff images. Tiff stacks can be converted with ImageJ with "File > Save As > Image Sequence" setting the option "Start At" to "1" and a hyphen needs to be added to the name to follow the naming convention <some text>-<FrameID>.tiff, where FrameID is a 4 digit leading zero-padded number, corresponding to the FrameID column in the feature table. Further details on this can be found in the R documentation for CellPhe's extractFeatures() function and can be accessed by running `?CellPhe::extractFeatures` in the R console.

The cell time series output by `extractFeatures()` can be saved as an RDS (R Data File) and used as input to the CellPhe GUI for interactive data exploration. Alternatively, the time series can be input to the function `varsFromTimeSeries()` which calculates variables that summarise the cells' behaviour over time, providing both summary statistics and indicators of time-series behaviour at different levels of detail obtained via wavelet analysis. The output is a dataframe that can be used in multivariate analysis, e.g. for classification or clustering.

The worked example below uses data from a PhaseFocus experiment. Additional guidance on how to import TrackMate input data is provided in section 4.6

```
library('CellPhe')

## define path to input feature table
trial_name <- "05062019_B3_3"
basedir <- "data"
input_feature_table <-  sprintf("%s/%s_Phase-FullFeatureTable.csv", basedir, trial_name)

## set the minimum number of frames a tracked cell should appear on
min_frames <- 50
```

```r
## get the frame and cell identifiers and ROI filenames and copy any required features
feature_table <- copyFeatures(input_feature_table, min_frames, source = "Phase")

## define the paths to ROI files and images
roi_folder <- sprintf("%s/%s_Phase", basedir, trial_name)
image_folder <- sprintf("%s/%s_imagedata", basedir, trial_name)

## calculate time series for all features
new_features <- extractFeatures(feature_table, roi_folder, image_folder, framerate = 0.0028)

## save RDS file for use in CellPhe GUI
saveRDS(new_features, file = "example.rds")

## calculate variables to characterise each feature's time series
tsvariables <- varsFromTimeSeries(new_features)

## save this output file, this is the same as the TreatedTraining.csv file described below
write.csv(tsvariables, "TreatedTraining.csv", row.names = FALSE)
```

Note that the worked example below consists of a data set of untreated cells and a data set of treated cells compiled to form a training set. The test set is formed in the same way with untreated and treated data sets independent of the training set. It would therefore be necessary to repeat the steps above for three further data sets to produce the training and test sets required for the worked example. Pre-compiled files called `UntreatedTraining.csv`, `TreatedTraining.csv`, `UntreatedTest.csv` and `TreatedTest.csv` are provided in the `example_data` folder to allow the user to follow the worked example without producing the outputs themselves.

4.3 Segmentation error prediction and removal

An optional step within the CellPhe workflow is the prediction of segmentation errors within a data set. This requires ground truth data sets of correctly segmented cells and those identified as segmentation errors. For accurate results, it will likely be necessary to compile new ground truth data sets for each cell type you work with due to heterogeneity between cell types. Ground truth data sets can be established by inspecting cells within image visualisation software such as ImageJ, noting cell IDs of correctly segmented cells and segmentation errors, and subsetting your feature table of extracted time series variables to only include these cells. Note that once ground truth data is available for a particular cell type, this can be re-used for further experiments involving the same cell type, including for example, different drug treatments. Ensure that you have one output file for correctly segmented cells and one for segmentation errors.

You will then be ready to predict segmentation errors in R. The CellPhe R package includes two functions for this: `predictSegErrors()` and `predictSegErrors_Ensemble()`. Both work by training a number of decision trees that are then used to predict whether or not a new set of cells contain any segmentation errors. Final classifications are made via a voting system, where a cell is classified as segmentation error if more than a defined proportion of decision trees predict it as such. `predictSegErrors_Ensemble()` adds further stringency to the prediction of segmentation errors by calling `predictSegErrors()` multiple times and a cell is given a final classification of segmentation error if it receives a vote for this class in at least half of the repeated runs. Both

functions output a list of the cells predicted as segmentation errors.

Feature tables of ground truth correctly segmented and erroneously segmented MDA-MB-231 cells are provided in `CorrectSegs_MDAMB231.csv` and `SegErrors_MDAMB231.csv` respectively. These files are used within the worked example below to train decision trees to recognise MDA-MB-231 cells that have been inaccurately segmented. We also provide two further data sets of correctly segmented and erroneously segmented MCF-7 cells, `CorrectSegs_MCF7.csv` and `SegErrors_MCF7.csv`. These files are not used within the worked example below but may be of use to users working with this cell line or wanting to form their own ground truth segmentation data sets.

```
## read in ground truth feature tables of correctly segmented cells and segmentation errors

correctsegs = read.csv("CorrectSegs_MDAMB231.csv", header = TRUE)
segerrors = read.csv("SegErrors_MDAMB231.csv", header = TRUE)
```

```
## read in data sets for segmentation error prediction

UntreatedTraining = read.csv("UntreatedTraining.csv", header = TRUE)
TreatedTraining = read.csv("TreatedTraining.csv", header = TRUE)
UntreatedTest = read.csv("UntreatedTest.csv", header = TRUE)
TreatedTest = read.csv("TreatedTest.csv", header = TRUE)

## add a column of true class labels to each data set

addGroupInfo<-function(dataset, group)
{
dataset<-cbind(rep(group, dim(dataset)[1]), dataset)
colnames(dataset)[1] = "Group"
return(dataset)
}

UntreatedTraining<-addGroupInfo(UntreatedTraining, "Untreated")
TreatedTraining<-addGroupInfo(TreatedTraining, "Treated")
UntreatedTest<-addGroupInfo(UntreatedTest, "Untreated")
TreatedTest<-addGroupInfo(TreatedTest, "Treated")

## form training and test sets

Training = rbind(UntreatedTraining, TreatedTraining)
Test = rbind(UntreatedTest, TreatedTest)
```

The `predictSegErrors_Ensemble()` function can then be used for segmentation error prediction. Default parameters are set as follows:

- `num`, number of decision trees to be trained = 50
- `K`, number of repeated runs of `predictSegErrors()` to be performed = 10

- `proportion`, proportion of votes needed for a final classification of segmentation error to be made = 0.7

but these can be customised to suit your own requirements.

The `removePredictedSegErrors()` function can be used to remove any identified segmentation errors prior to downstream analysis.

```
## identify segmentation errors within training and test sets

segErrors_Training<-predictSegErrors_Ensemble(segerrors, correctsegs, 50, 10, Training[,-1],
Training[,2], 0.7)

segErrors_Test<-predictSegErrors_Ensemble(segerrors, correctsegs, 50, 10, Test[,-1],
Test[,2], 0.7)

## remove predicted segmentation errors from training and test sets

Training<-removePredictedSegErrors(Training, 2, segErrors_Training)
Test<-removePredictedSegErrors(Test, 2, segErrors_Test)
```

Note that to reproduce the results of section 4.5 within the CellPhe GUI it would be necessary to export the test set containing cells from both `UntreatedTest.csv` and `TreatedTest.csv`, this is because the classification algorithm requires both classes to be present within the test set. This forms the `AllTest.csv` file used within the GUI demonstration video and `AllTestLabels.csv` is simply a column vector of true class labels for each cell.
Both of these files can be obtained with the following code:

```
write.csv(Test[,-1], "AllTest.csv", row.names = FALSE)
write.table(Test[,1], "AllTestLabels.csv", row.names = FALSE, col.names = FALSE)
```

4.4 Calculating separation scores

Separation scores can be calculated to identify discriminatory variables for feature selection. The higher the separation score, the better a variable is at discriminating between the two cell populations. The `calculateSeparationScores()` function can be used to obtain a table of separation scores. The output is a data frame, where the first column lists the variable indices, the second column lists the variable names and the third lists the separation scores. A threshold can be set to only display separation scores above a defined threshold, and the calculateOptimalThresh argument can be set to `TRUE` to determine the optimal separation threshold as described within the CellPhe paper. Note that the default threshold is 0 unless otherwise defined.

```
## subset training set into untreated and treated sets

UntreatedTraining<-subset(Training, Training$Group == "Untreated")
TreatedTraining<-subset(Training, Training$Group == "Treated")
```

To obtain the full list or separation scores:

```r
## calculate separation scores with default parameters

sepscores<-calculateSeparationScores(UntreatedTraining[,-c(1,2)], TreatedTraining[,-c(1,2)])
```

or to only store a list of separation scores above the optimal separation threshold:

```r
## calculate separation scores with calculateOptimalThresh = TRUE

sepscores<-calculateSeparationScores(UntreatedTraining[,-c(1,2)], TreatedTraining[,-c(1,2)],
calculateOptimalThresh = TRUE)
```

Separation scores can then be used for feature selection by subsetting the full training and test sets so that only variables with separation scores above the desired threshold are retained.

```r
## Subset training and test sets to only include features with separation scores above the
## selected threshold

Training = cbind(Training[ ,c(1,2)], Training[ ,sepscores[,2]])
Test = cbind(Test[ ,c(1,2)], Test[ ,sepscores[,2]])
```

4.5 Cell population ensemble classification

CellPhe's `cellPopulationClassification()` function can be used for training and testing of an ensemble of classifiers, namely Linear Discriminant Analysis (LDA), Random Forest (RF) and Support Vector Machines (SVM). The function outputs a table of predicted classes, with the first, second and third columns corresponding to predictions from LDA, RF and SVM respectively. The fourth column lists final predicted class labels for each cell based on a majority vote system.

R's `table()` function can be used to obtain a confusion matrix of ensemble classification results so that classification accuracy scores can be calculated.

```r
## Perform ensemble classification

classifications<-cellPopulationClassification(Training[,-c(1,2)], Test[,-c(1,2)],
as.factor(Training[,1]))

## Display confusion matrix of results

table(Real = Test[,1], Predicted = classifications[,4])
```

4.6 Trackmate input data

The following steps can be followed to read segmentation and tracking information obtained from TrackMate into CellPhe. Information on how to complete the preliminary segmentation and tracking steps can be found here. Once segmentation and tracking are complete, it is necessary to save a .csv file of tracking information and an RoiSet zip file. Visual demonstrations of how to obtain

such files are provided in **Figure ??**. Note that it is important to keep naming conventions for all input files consistent, as in the PhaseFocus example in section 4.2. Ensure image folder, the file of tracking information and the zipped ROI folder share consistent file names, for example ”05062019_B3_3_imagedata”, ”05062019_B3_3_Track.csv” and ”05062019_B3_3_RoiSet.zip”.

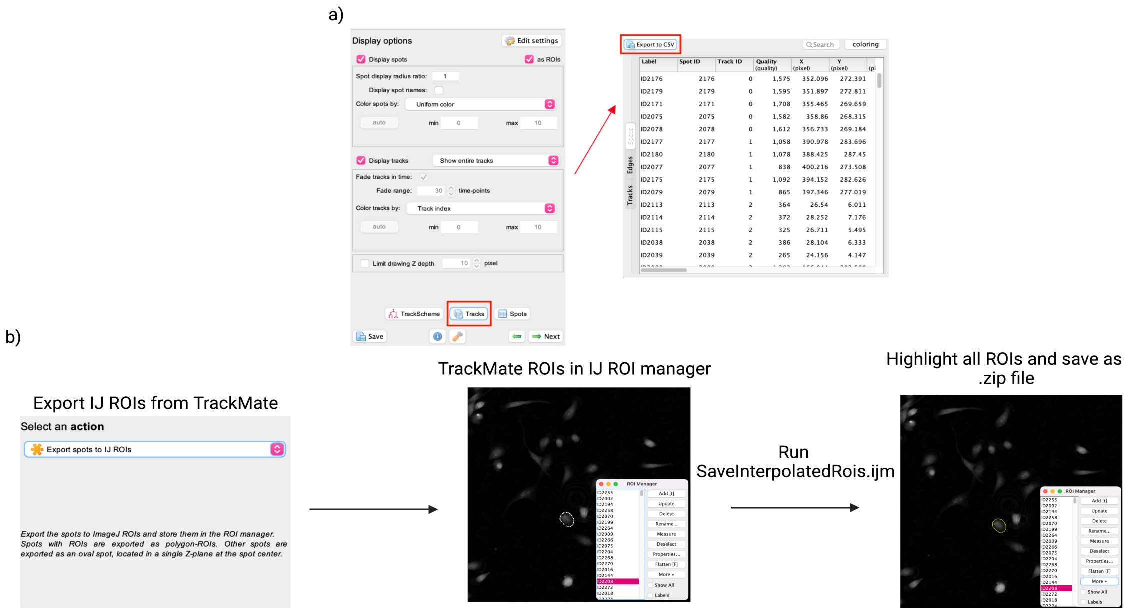

Supplementary Figure 5. a) Once segmentation and tracking are complete within ImageJ’s TrackMate plugin, the Tracks data table can be exported as a .csv file which will be used as input for CellPhe. **b)** All ROIs identified within the TrackMate plugin can be exported as ImageJ (IJ) ROIs and will then be displayed in the ROI manager. The SaveInterpolatedRois.ijm macro from https://github.com/uoy-research/CellPhe can then be ran for interpolation of cell boundaries. All ROIs can then be highlighted and saved as a zipped file.

Once all files are in the current R working directory and the RoiSet folder has been unzipped, the following code can be ran to extract time series variables, note the change in file names and the change from `source = "Phase"` to `source = "Track"`:

```r
library('CellPhe')

## define path to input feature table
trial_name <- "05062019_B3_3"
basedir <- "data"
input_feature_table <-  sprintf("%s/%s_Track.csv", basedir, trial_name)

## set the minimum number of frames a tracked cell should appear on
min_frames <- 50
## get the frame and cell identifiers and ROI filenames and copy any required features
feature_table <- copyFeatures(input_feature_table, min_frames, source = "Trackmate")
```

```r
## define the paths to ROI files and images
roi_folder <- sprintf("%s/%s_RoiSet", basedir, trial_name)
image_folder <- sprintf("%s/%s_imagedata", basedir, trial_name)

## calculate time series for all features
new_features <- extractFeatures(feature_table, roi_folder, image_folder, framerate = 0.0028)

## save RDS file for use in CellPhe GUI
saveRDS(new_features, file = "example.rds")

## calculate variables to characterise each feature's time series
tsvariables <- varsFromTimeSeries(new_features)

## save this output file, this is the same as the TreatedTraining.csv file described below
write.csv(tsvariables, "TreatedTraining.csv", row.names = FALSE)
```

5 CellPhe GUI

The interactive CellPhe GUI, accessed here: https://cellphegui.shinyapps.io/app_to_host/, provides a user-friendly platform for data exploration, cell type classification and identification of heterogeneous clusters. The GUI can be used to visualise cell time series and extract time series variables from feature tables in the same way as the `varsFromTimeSeries()` function, output files can then be explored in real-time or saved for future use. Discriminatory variables can be identified with ease through calculation of separation scores, beeswarm plots, PCA, UMAP and t-SNE. Furthermore, all plots are interactive, facilitating identification and isolation of single cells for further investigation. Training and test sets can be uploaded for ensemble classification, with options for supervised and unsupervised classification. Classification models can be validated within the GUI through confusion matrices, accuracy metrics and ROC curves. The CellPhe GUI also facilitates hierarchical and k-means clustering for identification of heterogeneous cell subsets. Clustering outputs are interactive, allowing the user to determine the optimal number of clusters within their data and explore feature importance.
