# Supplementary material for: The CellPhe toolkit for cell phenotyping using time-lapse imaging and pattern recognition
Source: Nat Commun. 2023 Apr 3;14:1854. doi: 10.1038/s41467-023-37447-3 (PMC10070448; doi:10.1038/s41467-023-37447-3)
Supplement: Supplementary file 3 — Reporting Summary [file 41467_2023_37447_MOESM3_ESM.pdf]

nature portfolio

Corresponding author(s): Julie Wilson

Last updated by author(s): Mar 8, 2023

Reporting Summary

Nature Portfolio wishes to improve the reproducibility of the work that we publish. This form provides structure for consistency and transparency in reporting. For further information on Nature Portfolio policies, see our Editorial Policies and the Editorial Policy Checklist.

Statistics

For all statistical analyses, confirm that the following items are present in the figure legend, table legend, main text, or Methods section.

n/a	Confirmed	
[ ]	[x]	The exact sample size (*n*) for each experimental group/condition, given as a discrete number and unit of measurement
[x]	[ ]	A statement on whether measurements were taken from distinct samples or whether the same sample was measured repeatedly
[ ]	[x]	The statistical test(s) used AND whether they are one- or two-sided<br>*Only common tests should be described solely by name; describe more complex techniques in the Methods section.*
[ ]	[x]	A description of all covariates tested
[x]	[ ]	A description of any assumptions or corrections, such as tests of normality and adjustment for multiple comparisons
[ ]	[x]	A full description of the statistical parameters including central tendency (e.g. means) or other basic estimates (e.g. regression coefficient) AND variation (e.g. standard deviation) or associated estimates of uncertainty (e.g. confidence intervals)
[ ]	[x]	For null hypothesis testing, the test statistic (e.g. *F*, *t*, *r*) with confidence intervals, effect sizes, degrees of freedom and *P* value noted<br>*Give P values as exact values whenever suitable.*
[x]	[ ]	For Bayesian analysis, information on the choice of priors and Markov chain Monte Carlo settings
[ ]	[x]	For hierarchical and complex designs, identification of the appropriate level for tests and full reporting of outcomes
[x]	[ ]	Estimates of effect sizes (e.g. Cohen's *d*, Pearson's *r*), indicating how they were calculated

Our web collection on statistics for biologists contains articles on many of the points above.

Software and code

Policy information about availability of computer code

Data collection	PhaseFocus Acquire V3.2.2 software was used for image acquisition. PhaseFocus Analyse V3.1.1 was used for image and raw feature table exportation, and for automated segmentation and tracking of cells. Trackmate v7.7.2 was also used for segmentation and tracking.
Data analysis	All feature extraction, time series analysis, classification and clustering was performed in RStudio, V 1.2.5042.<br>R packages used were tiff v0.1-11, stats v4.1.3, smotefamily v1.3.1, randomForest v4.7-1.1, e1071 v1.7-12, factoextra v1.0.7, tree v1.0-42, devtools v2.4.3.<br>The R package for CellPhe (version 0.0.0.9000) is publicly available on Github and can be accessed via Zenodo: https://zenodo.org/record/7620171\#.ZAJZMuzP0o8.<br>Prism V 9.1.0 (216) was used to produce barcharts and beeswarm plots included in this study.<br>ImageJ v2.9.0-1.53t was used to interpolate ROI boundaries obtained from Trackmate.<br>A graphical user interface (GUI) is hosted at https://cellphegui.shinyapps.io/app_to_host/ and a video demonstrating use of the GUI is available on Zenodo: https://zenodo.org/record/7674584\#.ZAJYBOzP0o8.

For manuscripts utilizing custom algorithms or software that are central to the research but not yet described in published literature, software must be made available to editors and reviewers. We strongly encourage code deposition in a community repository (e.g. GitHub). See the Nature Portfolio guidelines for submitting code & software for further information.

Data

Policy information about availability of data

All manuscripts must include a data availability statement. This statement should provide the following information, where applicable:

- Accession codes, unique identifiers, or web links for publicly available datasets
- A description of any restrictions on data availability
- For clinical datasets or third party data, please ensure that the statement adheres to our policy

All data used to produce the results in the manuscript, including separate data that will allow the user to follow the worked example in the CellPhe user guide, are available from the Dryad database: https://doi.org/10.5061/dryad.4xgxd25f0. Here, the file example_data.zip contains all the data required to follow the worked example and a video that explains how to use the GUI is available on Zenodo: https://zenodo.org/record/7674584\#.ZAJYBOzP0o8. Source data are provided with this paper.

Human research participants

Policy information about studies involving human research participants and Sex and Gender in Research.

Reporting on sex and gender	Reporting on sex and gender is not applicable.
Population characteristics	No human research participants were involved.
Recruitment	No participants were recruited.
Ethics oversight	Not applicable.

Note that full information on the approval of the study protocol must also be provided in the manuscript.

Field-specific reporting

Please select the one below that is the best fit for your research. If you are not sure, read the appropriate sections before making your selection.

☒ Life sciences ☐ Behavioural & social sciences ☐ Ecological, evolutionary & environmental sciences

For a reference copy of the document with all sections, see nature.com/documents/nr-reporting-summary-flat.pdf

Life sciences study design

All studies must disclose on these points even when the disclosure is negative.

Sample size	Statistical methods were not used to predetermine sample size as there are no effect sizes or predetermined proportions involved in our study. However, cell seeding densities (i.e. the number of cells per well) were kept consistent throughout all experiments to ensure reproducibility and were guided by the user manual provided by PhaseFocus. Furthermore, balanced training sets were used to ensure no bias towards training of one class over the other.
Data exclusions	To ensure reliable characterisation of cellular phenotype, only cells that were tracked for 50 frames or more were included in analyses. Furthermore, our toolkit includes automated identification and exclusion of segmentation errors prior to downstream analyses.
Replication	Training sets were a compilation of experiments performed in different months to increase sample size for training and to ensure that identified discriminatory variables were not a result of experimental variability. Independent test sets were used for model testing where data included in test sets were from experiments carried out by a different individual, and test set data was not used during model training. Findings were consistent across multiple experiments and models achieved high classification accuracy for independent test sets. The CellPhe method was validated using further experiments with different cell lines and different drugs.
Randomization	Data sets for entire experiments were randomly assigned as either training or test data so that cells from the same experiment were never assigned to both training and test sets.
Blinding	Blinding was not possible as cell cultures were either treated or not treated and therefore cells could not be allocated to groups randomly. During training, supervised machine learning algorithms require class labels to be associated with training data, but independent test sets, never used in model training, were used to assess model performance and therefore avoid bias. Test set labels were only used to calculate the accuracy of classification.

Reporting for specific materials, systems and methods

We require information from authors about some types of materials, experimental systems and methods used in many studies. Here, indicate whether each material, system or method listed is relevant to your study. If you are not sure if a list item applies to your research, read the appropriate section before selecting a response.

Materials & experimental systems

n/a	Involved in the study
☒	☐ Antibodies
☐	☒ Eukaryotic cell lines
☒	☐ Palaeontology and archaeology
☒	☐ Animals and other organisms
☒	☐ Clinical data
☒	☐ Dual use research of concern

Methods

n/a	Involved in the study
☒	☐ ChIP-seq
☒	☐ Flow cytometry
☒	☐ MRI-based neuroimaging

Eukaryotic cell lines

Policy information about cell lines and Sex and Gender in Research

Cell line source(s)	MCF-7 and MDA-MB-231 cells used within this study were originally obtained from the American Type Culture Collection (ATCC).<br>(https://www.atcc.org/).
Authentication	Molecular identity was confirmed by short tandem repeat analysis (Masters et al., 2001).
Mycoplasma contamination	Cells were confirmed to be mycoplasma-free by 40,6-diamidino-2-phenylindole (DAPI) method (Uphoff et al., 1992).
Commonly misidentified lines<br>(See ICLAC register)	No commonly misidentified cell lines were used within this study.
